# A Mathematical Prodigy

**Published:** 1888-02

**Authors:** 


### A MATHEMATICAL PRODIGY.

Of "lightning calculators" there is always a fair supply. These may be produced artificially. There are certainly short-cut methods of calculation which any one with a good memory and a quick perception, may acquire, and with a little facility in the use of these abbreviated methods a common order of intellect may perform feats which easily dazzle those who have paid no particular attention to subjects of that sort. But in Evening School No. 58, in Fifty-second street, near Eight avenue, is a mathematical genius who is not a product of art.

William Ulysses Scott lives with his father at 743 Sixth avenue. He was born in Hoboken, N. J., seventeen years ago. It was at Public School No. 8, in Jersey City, that the boy's peculiar gift was first noticed. In 1883, the family came to New York. In September, he presented himself at Grammar School No. 69, in Fifty-fourth street, which is presided over by Matthew G. Elgas. He was admitted into the class next the highest. Nothing unusual was said or noticed about the boy for some time. He is very quiet and modest, and would be the last to herald his talent. The class teacher noticed that when the boys were "doing" arithmetic Scott always had his answers first, but never could show any work or tell how he got the result. He was suspected of copying from his neighbors; but, watch as he might, the teacher could not detect the cheat, if it was a cheat. Whenever the work and the explanation were required, Scott could count on having a "failure" against him on the record.—*New York Times.*

